# A Systematic Review and Meta‐Analysis Examining the Effect of Mindfulness Based Stress Reduction on Pain Severity and Quality of Life in People Living With Fibromyalgia

**DOI:** 10.1002/ejp.70239

**Published:** 2026-03-29

**Authors:** Edward Walsh, Kayleigh Hart, Bettina Forster

**Affiliations:** ^1^ City and St. George's, University of London London UK; ^2^ Independent Researcher

**Keywords:** chronic pain, meditation, persistent pain

## Abstract

**Background and Objective:**

This systematic review and meta‐analysis investigated the effect of Mindfulness Based Stress Reduction (MBSR) on pain severity, quality of life, pain catastrophising, and depression for people living with Fibromyalgia (FM) at short and long term follow up.

**Databases and Data Treatment:**

MEDLINE, EMBASE, CENTRAL, ClinicalTrials.gov, WHO Trial Registry, CINAHL and PsychInfo were searched from inception to December 2025 for English language full papers. Randomised and non‐randomised trials were included where MBSR was compared with no treatment, usual care or any active control; online MBSR interventions were excluded.

**Results:**

The search identified 566 records, of which 11 original trials and 1153 participants were included, 1097 of whom were women. A predefined risk of bias tool was used to assess included studies. Fixed effect model meta‐analysis showed improvements in favour of MBSR compared with active controls at long term follow up in quality of life (SMD −0.2635 [95% CI −0.4725, −0.0545]) and pain catastrophising (SMD −0.5375 [95% CI −0.8323, −0.2428]). Significant effects on pain severity (SMD −0.2966 [95% CI −0.4939, −0.0992]) and depression (SMD −0.4452 [95% CI −0.6502, −0.2402]) were only present at short term follow up versus passive control. Grading of Recommendations Assessment, Development and Evaluation (GRADE) determined the certainty of outcomes ranging from very low to moderate.

**Conclusions:**

MBSR improves pain catastrophising and quality of life in people with FM at short and long term follow up; pain severity and depression were not significantly alleviated versus active control. OSF Registration: DOI 10.17605/OSF.IO/TJ5HX.

**Significance Statement:**

This meta‐analysis reveals that, among individuals living with fibromyalgia, mindfulness based stress reduction does not significantly reduce pain severity, updating the guidance from the last review in 2013. Mindfulness based stress reduction does however have a small positive effect on quality of life compared with other active treatments, at both short and long term follow up. This suggests there is a long lasting, mindfulness specific mechanism that improves quality of life.

## 1 Introduction

Mindfulness Based Stress Reduction (MBSR) was developed to address persistent pain by modulating one's relationship to their inner experience (Kabat‐Zinn J., 2005). The last systematic review specifically examining the effect of Mindfulness Based Stress Reduction (MBSR) on Fibromyalgia (FM) is over a decade old (Lauche et al. 2013). A weak recommendation was made in favour of MBSR for quality of life and pain severity outcomes, limited by a dearth of high quality trials. The top research priority for patients, clinicians and funders, is identifying the most effective treatment strategies (James Lind Alliance Priority Setting Partnership, n.d.). This review provides an updated examination of the literature exploring the efficacy of MBSR for the treatment of FM symptoms.

The FM pathoaetiology can perhaps best be understood within the biopsychosocial model (Popkirov et al. 2020), initially proposed in relation to psychiatric diagnoses by Engels and later adapted for non‐psychiatric diagnoses like FM (Engel 1981; Kusnanto et al. 2018). It proposes that a confluence of biological, psychological and social factors needs to be considered when considering the drivers for an individual's pain experience (Bolton 2022). This is helpful in understanding FM, which is typified by a lack of proportionate identified biological factors that explain symptom duration and intensity (Perrot 2019).

Mindfulness‐Based Stress Reduction (MBSR) entails an intensive 8 week programme with at least 2 h long weekly sessions and an intensive retreat, typically for a day (Carmody and Baer 2009). The MBSR programme includes an array of mindfulness practices including sitting meditation, walking meditation and yoga (Kabat‐Zinn 2005). The sessions aim to cultivate awareness of one's sensations, emotions and thoughts as they are experienced in the present (Day et al. 2014). Seminal research on mindfulness based interventions showed increased physical activity levels and reduced analgesia utilisation when taught to people with chronic pain (Kabat‐Zinn et al. 1985).

The last meta‐analysis to specifically investigate MBSR for FM was conducted in 2013, and found reduced pain severity and improved quality of life when compared with active control and usual care groups (Lauche et al. 2013). A more recent review investigating mindfulness based interventions including MBSR and Acceptance and Commitment Therapy (ACT) for FM found moderate effects favouring mindfulness interventions over control conditions for pain, anxiety, depression, mindfulness, sleep quality and quality of life (Haugmark et al. 2019); however although ACT and MBSR share a mindfulness focus, they are distinct interventions, shown to produce different effects in populations living with chronic pain (Veehof et al. 2016). No current or underway systematic reviews or scoping reviews on the topic were identified, an updated review was therefore warranted.

This systematic review investigates the literature up to December 6th 2025 detailing the impact of MBSR on FM at short and long term follow up and adds a meta‐analysis providing a clear quantitative assessment. The primary objective of the review is to determine the effect of MBSR on pain intensity and quality of life in FM. As secondary outcomes, MBSR effects on pain catastrophising and depression were investigated.

## 2 Literature Search Methods

The systematic review and meta‐analysis was conducted in accordance with Cochrane Handbook (Cumpston et al. 2019), Cochrane Musculoskeletal Group (Ghogomu et al. 2014), Preferred Reporting Items for Systematic reviews and Meta‐Analyses (PRISMA) (Page et al. 2021) and Grading of Recommendations Assessment, Development and Evaluation (GRADE) (Guyatt et al. 2008) recommendations. The protocol was registered on the Open Science Framework prior to study commencement (Walsh and Forster 2024).

### 2.1 Eligibility Criteria

We included both randomised controlled trials and non‐randomised trials with active and waiting list control groups. Only published, fully complete studies were included. Conference abstracts and studies lacking all outcomes of interest were excluded.

Studies investigating cohorts with mixed diagnoses were included if at least 50% of participants had FM or if FM was the most common participant condition. No age, sex, or geographic criteria needed to be met for inclusion, and there was no FM specific diagnostic criteria or severity related restriction. MBSR research was included where MBSR was between 6 and 10 group sessions of 2–4 h with the cultivation of mindfulness as the key element. Studies with secondary co‐interventions were included.

Studies not written in English were excluded for practicality reasons and MBSR interventions conducted online were excluded since virtual delivery of MBSR presents a significant extraneous variable. Effects were categorised as short term (directly after the intervention) and long term (the latest recorded follow up data, at least 3 months post randomisation).

### 2.2 Search Strategy

**FIGURE 1:**
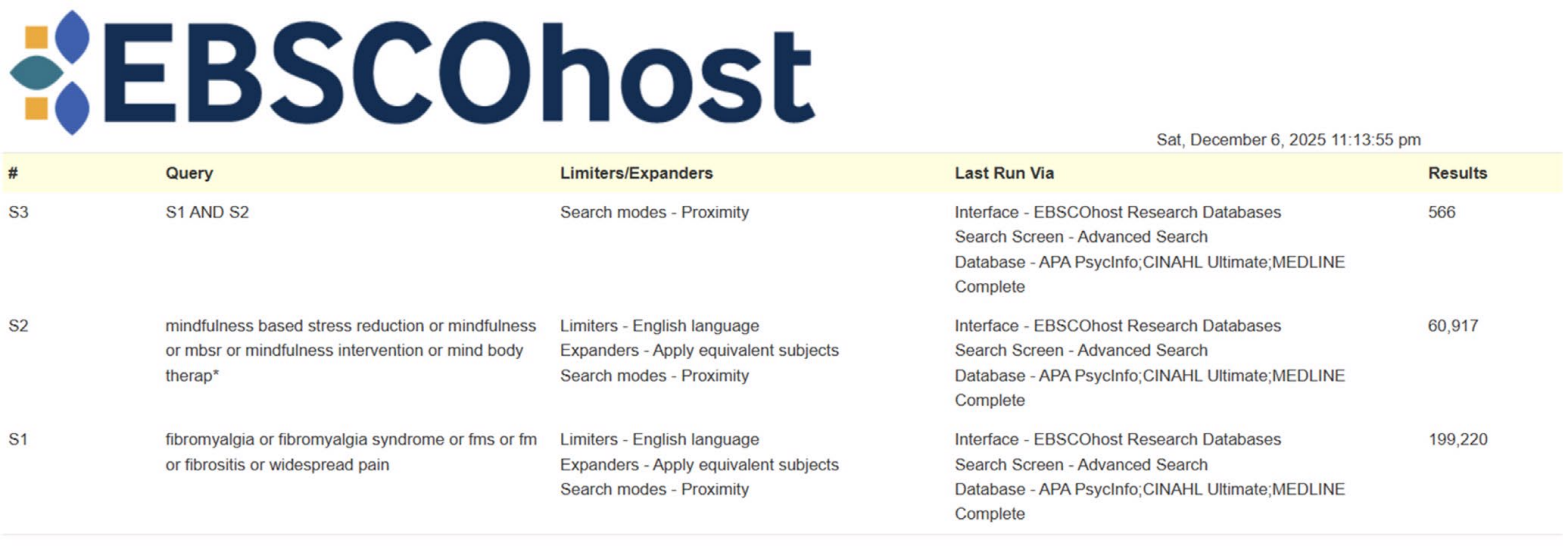
Search strategy. Systematic search strategy detailing the search terms, search modes, databases searched and number of results.

A three‐step search strategy was utilised in this review. First, an initial limited search of MEDLINE (PubMed) and CINAHL (EBSCO) was undertaken to identify articles on the topic. The text words contained in the titles and abstracts of relevant articles and the index terms used to describe the articles were used to develop a full search strategy for EBSCO (see Figure 1). The search strategy, including all identified keywords and index terms, was adapted for each included database. The reference list of all included sources of evidence was screened for additional studies. References of systematic reviews with the same or a similar question were also screened for additional studies.

Studies published in English were included. The databases searched from inception to December 6th 2025 are as follows: MEDLINE, EMBASE, CENTRAL, ClinicalTrials.gov, WHO Trial Registry, CINAHL and PsychInfo.

### 2.3 Source of Evidence Selection

**FIGURE 2:**
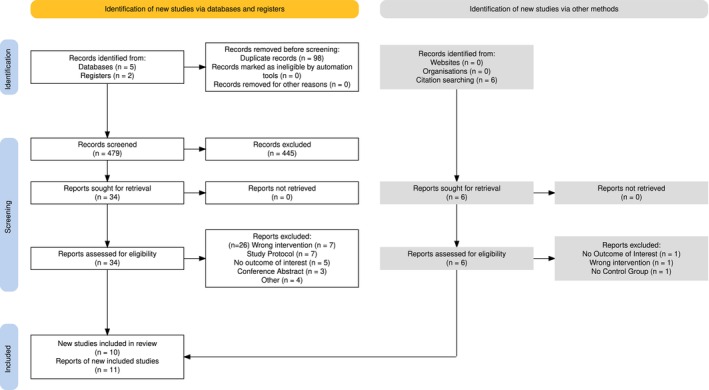
PRISMA flow diagram. Diagrammatic representation of study identification, screening and inclusion process with reasons given for report exclusion during eligibility assessment.

Following the search, all identified articles were collated and uploaded into Rayyan (Ouzzani et al. 2016) and duplicates removed. Following a pilot test, titles and abstracts were then screened by two independent reviewers for assessment against the inclusion criteria for the review. Following title and abstract screening, potentially relevant sources were retrieved in full and their citation details imported into Zotero (Takats et al. 2023). The full text of selected citations was assessed in detail against the inclusion criteria by two independent reviewers. Reasons for exclusion of sources of evidence at full text review that did not meet the inclusion criteria were recorded and reported. One reviewer collected the data from each report into a table, which was subsequently checked by the second reviewer. Any disagreements that arose between the reviewers at each stage of the selection process were resolved through discussion. The results of the search and the study inclusion process are reported in the Figure 2 below (Haddaway et al. 2022; Page et al. 2021).

### 2.4 Data Items

Pain severity, quality of life, pain catastrophising and depression outcomes were collected at short and long term follow up end points for the intervention group, active control group and waiting list control group for each report. All results that were compatible with each outcome domain in each study were sought. The number of participants, how many participants of each sex, and how many participants had a diagnosis of FM within each study were recorded. Details about the specifics of the MBSR intervention, the instructor(s), and the time point for long term follow up outcome measurement were also recorded. All the included studies had clearly outlined data variables of interest recorded.

### 2.5 Methodological Quality Assessment

The risk‐of‐bias tool was implemented as determined by the updated method guidelines for Cochrane musculoskeletal group systematic reviews and meta analyses (Ghogomu et al. 2014). It was used by the two independent assessors to determine the following areas each rated low, high, or unclear risk: (1) randomisation sequence generation; (2) allocation concealment; (3) blinding of participants and personnel; (4) blinding of outcome assessment; (5) incomplete outcome data; (6) selective outcome reporting; and (7) “other sources of bias”. Other sources of bias considered funding bias or other major methodological flaws.

**FIGURE 3:**
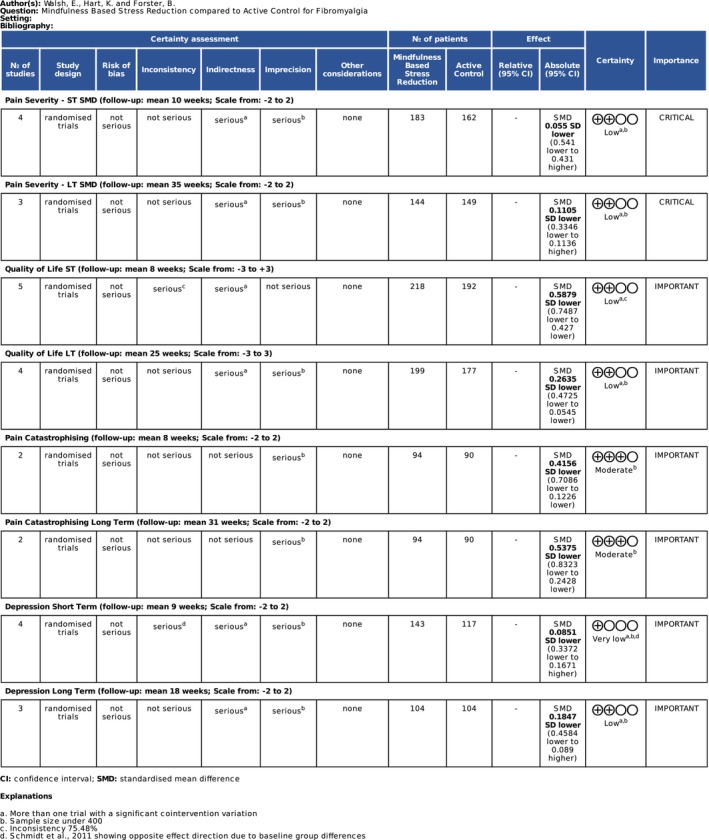
GRADE evidence profile. Grading of Recommendations Assessment, Development and Evaluation certainty assessment considering inconsistency, indirectness and imprecision.

GRADE criteria (Guyatt et al. 2008) were used to classify the certainty of the available evidence for MBSR versus active control for each outcome of interest at short and long term follow up (Figure 3).

### 2.6 Statistical Analysis

**FIGURE 4:**
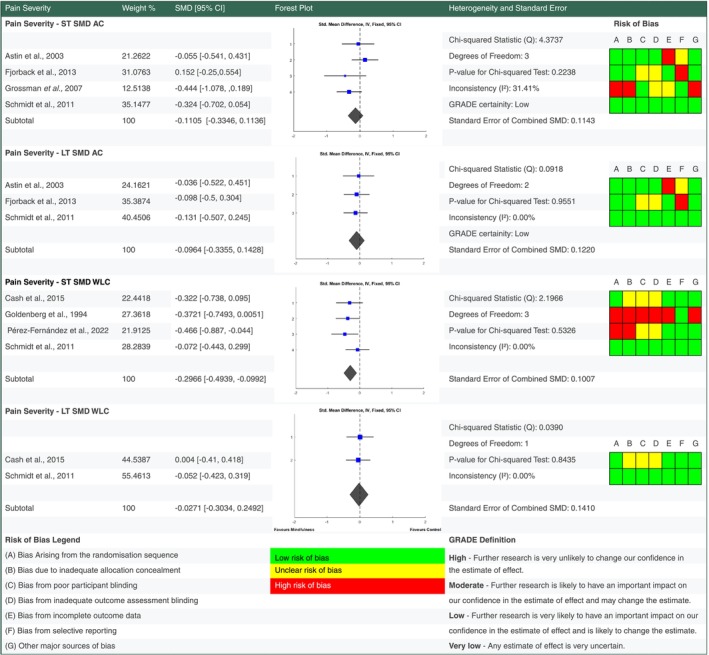
Forest plot for meta‐analyses of effects of mindfulness based stress reduction on pain severity. Details of percentage study weights, standardised mean difference outcomes with 95% confidence intervals, a forest plot and further fixed effect meta‐analysis statistic outcomes, alongside risk of bias assessments for pain severity findings at short and long term follow up versus active control and waiting list control groups. Grading of Recommendations Assessment, Development and Evaluation (GRADE) evidence certainty outcomes are also provided for short and long term outcomes versus active control. AC, active control; GRADE, grading of recommendations assessment, development and evaluation; LT, long term follow up; ST, short term follow up.

**FIGURE 5:**
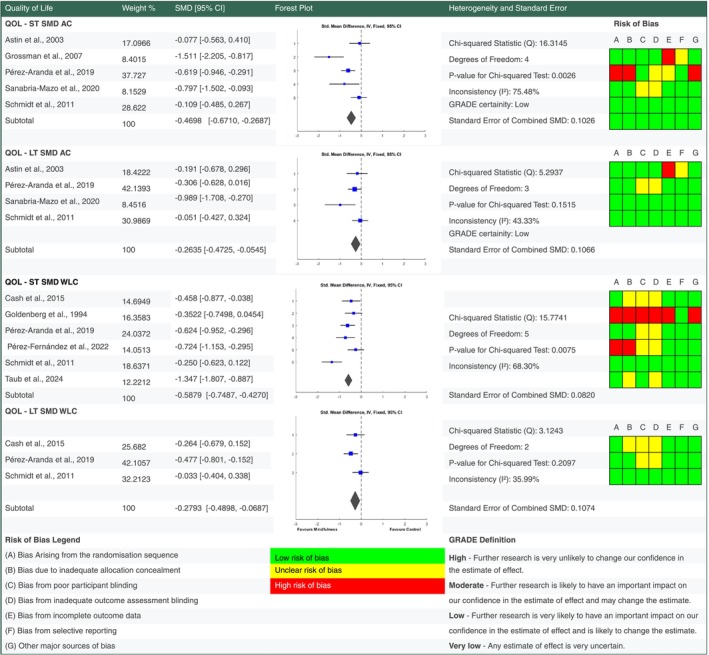
Forest plot for meta‐analyses of effects of mindfulness based stress reduction on quality of Life. Details of percentage study weights, standardised mean difference outcomes with 95% confidence intervals, a forest plot and further fixed effect meta‐analysis statistic outcomes, alongside risk of bias assessments for quality of life findings at short and long term follow up versus active control and waiting list control groups. Grading of Recommendations Assessment, Development and Evaluation (GRADE) evidence certainty outcomes are also provided for short and long term outcomes versus active control. AC, active control; GRADE, grading of recommendations assessment, development and evaluation; LT, long term follow up; ST, short term follow up.

**FIGURE 6:**
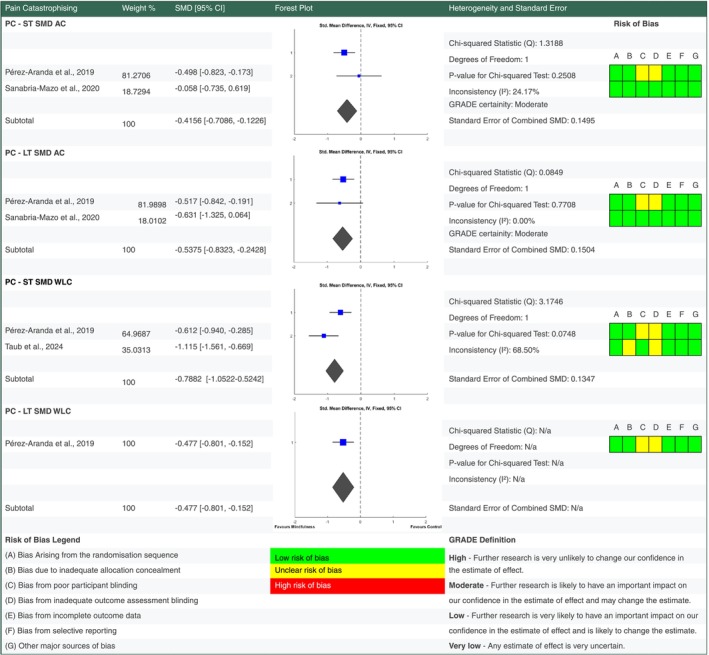
Forest plot for meta‐analyses of effects of mindfulness based stress reduction on pain catastrophising. Details of percentage study weights, standardised mean difference outcomes with 95% confidence intervals, a forest plot and further fixed effect meta‐analysis statistic outcomes, alongside risk of bias assessments for pain catastrophising findings at short and long term follow up versus active control and waiting list control groups. Grading of Recommendations Assessment, Development and Evaluation (GRADE) evidence certainty outcomes are also provided for short and long term outcomes versus active control. AC, active control; GRADE, grading of recommendations assessment, development and evaluation; LT, long term follow up; ST, short term follow up.

**FIGURE 7:**
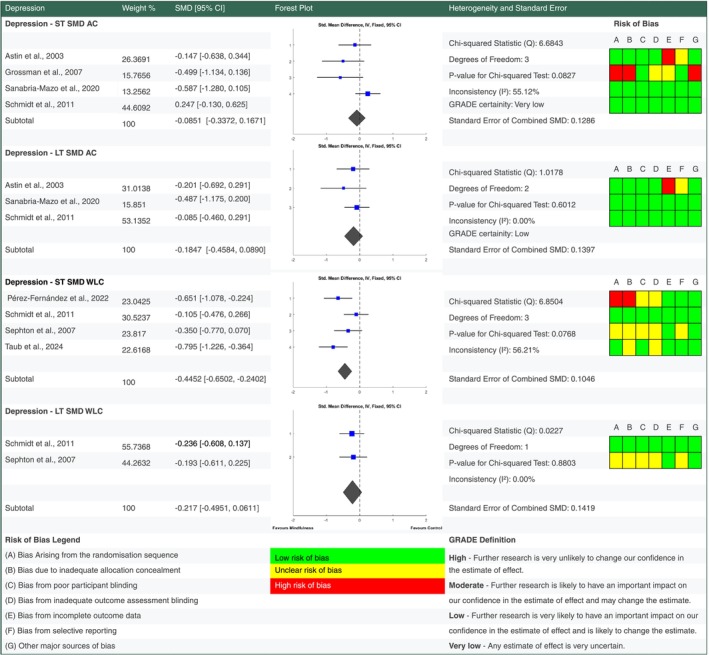
Forest plot for meta‐analyses of effects of mindfulness based stress reduction on depression. Details of percentage study weights, standardised mean difference outcomes with 95% confidence intervals, a forest plot and further fixed effect meta‐analysis statistic outcomes, alongside risk of bias assessments for depression findings at short and long term follow up versus active control and waiting list control groups. Grading of Recommendations Assessment, Development and Evaluation (GRADE) evidence certainty outcomes are also provided for short and long term outcomes versus active control. AC, active control; GRADE, grading of recommendations assessment, development and evaluation; LT, long term follow up; ST, short term follow up.

For each outcome standardised mean change scores were calculated from baseline to short term and long term follow up end points (see supplementary data for analysis). Standardised mean difference scores were also calculated for intervention outcomes versus active control and waiting list control at short and long term follow up (Figures 4, 5, 6, 7). Study eligibility for each synthesis was determined by tabulating study intervention characteristics and comparing against the pre‐planned eligibility criteria outlined above. Results of individual studies were also tabulated for each outcome prior to conversion to standardised outcomes. No required summary statistics were missing in the included studies.

**FIGURE 8:**
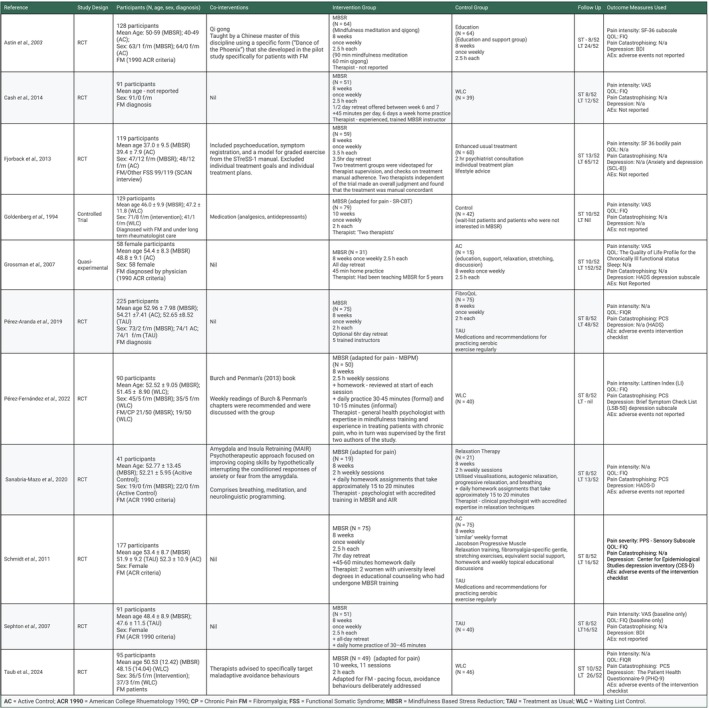
Included study characteristics. Tabulated study details including study design, population, intervention type and length, and the outcome measures used. AC, active control; ACR 1990, American college rheumatology 1990; CP, chronic pain; FM, fibromyalgia; FSS, functional somatic syndrome; MBSR, mindfulness based stress reduction; TAU, treatment as usual; WLC, waiting list control.

**FIGURE 9:**
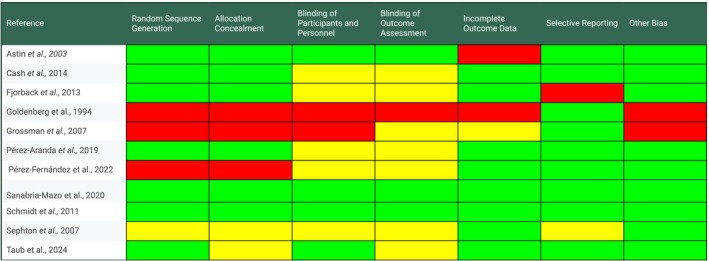
Risk of bias summary. A breakdown of the risk of bias for each included study based on the presence or absence of random sequence generation, allocation concealment, blinding of participants and personnel, blinding of outcome assessment, incomplete outcome data, selective reporting and other bias. Other bias considered funding bias or other major methodological flaws. Red denotes a high risk of bias, yellow an unclear risk and green a low risk.

We conducted the meta‐analysis using a fixed effect model using the inverse‐variance method, chosen due to the homogeneity of the population being studied and specificity of the MBSR intervention (Deeks et al. 2023). Fixed effect models are also more appropriate when investigating relatively small groups and weights, which were anticipated prior to the search commencement based on prior reviews and due to the division of outcomes by time end point and type of control comparator (Nikolakopoulou et al. 2014).

In all studies, controls included were waiting list control groups and active control groups. Because different scales were used to measure the same outcomes, we computed standardised mean differences (SMDs) between active control and MBSR groups. Outcome measures with inverse directions of effect had their means multiplied by −1 to ensure uniform outcome effect direction prior to standardised mean difference calculation.

MATLAB (The MathWorks Inc., n.d.) was used to calculate study weights, SMD's and to create forest plots (see supplementary MATLAB Code) for each outcome of interest: pain severity, quality of life, pain catastrophising and depression. Statistical heterogeneity was also evaluated in Matlab using chi‐squared and inconsistency was calculated using *I* ^2^ to estimate the percentage of effect estimate variability that relates to statistical heterogeneity as opposed to sampling error. No subgroup analysis was conducted due to the consistency of population and standardised intervention being investigated.

## 3 Results

### 3.1 Study Selection

34 of 566 articles were identified as potentially eligible and screened in full‐text (Figure 2). 11 trials met the inclusion criteria.

Studies that initially appeared to meet inclusion criteria but were ultimately excluded included a pilot cohort study published in 2017 (Ali et al. 2017) as less than half of the 15 participants had Fibromyalgia. A quasi‐randomised study from 2009 was excluded on the grounds the intervention was Body Mind Awareness—based on MBSR but distinct (Sampalli et al. 2009). Finally, a 1997 study was excluded as there was no control group (Kaplan et al. 1993).

### 3.2 Trial Characteristics

The characteristics of the study samples, interventions, co‐interventions, control groups, follow up time points and outcome measures are shown in Figure 8.

The included trials were published between 1994 and 2024 and had a total of 1153 participants. Two trials (Schmidt et al. 2011; Cash et al. 2015) analysed different outcomes from the same study population; both trials were therefore included with the participants from each counted only once.

Five of the trials investigated MBSR as a stand‐alone intervention. The other six trials combined MBSR with various co‐interventions (Qi gong; psychoeducation and graded exercise from a non‐individualised manual; analgesics and antidepressants; weekly reading of a practical mindfulness for health book; ‘Amygdala and Insula retraining’ comprising breathwork, meditation and neurolinguistic programming; and therapists instructed to target maladaptive avoidance behaviours).

Five trials compared MBSR with a waiting list control or treatment as usual only. The other six used a variety of active controls (education and a support group; usual treatment enhanced with psychiatric consultation and an individualised treatment plan; education, a support group with stretching and relaxation; ‘FibroQoL’; relaxation therapy with visualisations, breathwork, relaxation techniques and homework; ‘Jacobson Progressive Muscle Relaxation’ training, education, stretching and social support). All the active control conditions mirrored the 8 week, two to two and a half hour weekly session MBSR structure aside from Fjorback et al. (2013), who used a single 2 h psychiatric consultation to enhance treatment as usual.

Short term follow up length varied between 8 and 13 weeks post intervention initiation, whilst long term follow up varied between 12 and 152 weeks post intervention initiation, with an overall mean average of 41 weeks post randomisation data collection for long term follow up.

### 3.3 Risk of Bias in Individual Studies

Risk of bias is shown above in Figure 9. Green indicates low risk of bias, yellow indicates unclear risk of bias, and red indicates high risk of bias.

Risk of selection bias is low in six studies, mixed in one study, unclear in one study, and high in three studies. Risk of blinding related bias is low in three studies, unclear in five studies, mixed in two studies, and high in one study.

### 3.4 Health Effects

When compared to an active control there is low certainty evidence MBSR improved QOL at short term (SMD −0.4698 [95% CI −0.6710, −0.2687]) and long term (SMD −0.2635 [95% CI −0.4725, −0.0545]) follow up in people living with FM. MBSR also improved pain catastrophising at short (SMD −0.4156 [95% CI −0.7086, −0.1226]) and long term (SMD −0.5375 [95% CI −0.8323, −0.2428]) follow up with moderate certainty. There is low to very low certainty evidence that MBSR had no significant pooled effect on depression and pain severity versus an active control at short and long term follow up.

Versus a waiting list control MBSR had a small benefit on pain severity at short (SMD −0.2966 [95% CI −0.4939, −0.0992]) but not long term (SMD −0.0271 [95% CI −0.3034, 0.2492]) follow up. Depression showed a significant improvement versus waiting list control at short term (SMD −0.4452 [95% CI −0.6502, −0.2402]) but not long term (SMD −0.217 [95% CI −0.4951, −0.0611]) follow up.

The GRADE process was established to ensure consistency in evidence quality ratings and the strength of recommendations based on research (Guyatt et al. 2008). The process was used for each outcome of interest at both short and long term follow up. Every outcome except quality of life at short term follow up lost a certainty grade due to a sample size of under 400 for the outcome in question introducing serious imprecision. Pain catastrophising at short and long term follow up was the only outcome not to also lose a certainty grade as a result of co‐intervention variation introducing serious indirectness to the outcome.

## 4 Discussion

This meta‐analysis found moderate certainty evidence for moderate positive effects of MBSR on pain catastrophising and low certainty evidence for small positive effects on quality of life at short and long term follow up versus active control groups. No significant difference was found when comparing MBSR and active controls on depression and pain severity outcomes, although the certainty of these effect estimates ranged from low to very low. Moderate short term improvements in depression symptoms and small short term pain severity improvements were present when MBSR was compared with a waiting list control.

Whilst there were short term improvements in pain severity versus waiting list control, no significant changes were found versus an active control group. This finding mirrors findings from a meta‐analysis of MBSR for low back pain (Anheyer et al. 2017), which showed versus usual care the MBSR mean effect was −0.96 points on a numerical rating scale [95% CI, −1.64 to −0.34 points]. This is less than the minimum clinically meaningful difference of −1.65 (Bahreini et al. 2020). Based on these findings, recommending MBSR for reducing pain severity in FM is not justified.

In contrast, quality of life improved following MBSR at both short and long term outcome measurement versus an active control. The benefit versus active control is worth noting as it suggests an MBSR specific mechanism for improving quality of life. Interestingly, two of these studies (Astin et al. 2003; Schmidt et al. 2011) found no significant change in pain severity at short or long term follow up despite the quality of life improvement, consistent with other literature showing pain intensity is not an independent correlate of quality of life in FM (Offenbaecher et al. 2021). Cochrane review findings evidence a clinically meaningful effect of mixed exercise on health related quality of life in people living with Fibromyalgia (Bidonde et al. 2023), suggesting combining MBSR and mixed exercise may be of even greater benefit.

Although only two of the included studies (Pérez‐Aranda et al. 2019; Sanabria‐Mazo et al. 2020) investigated pain catastrophising, neither scored highly for risk of bias in any category, improving the robustness of the moderate effect size improvement finding at short and long term follow up versus active control. Recent research shows baseline pain catastrophising is prognostic of pain severity change and that changes in pain catastrophising are associated with pain severity change in FM (Paschali et al. 2021; Angst et al. 2022), however, neither study in this review that investigated pain catastrophising also investigated pain severity.

Whilst there were short term improvements in depression versus waiting list control, no significant changes were found versus an active control group. A lack of depression effect versus active control is consistent with findings from Lauche et al. (2013), despite more recent cross sectional data suggesting a protective effect of mindfulness on depressive symptoms in people living with FM Brooks et al. (2020). Qualitative research describes applied mindfulness causing a shift in depression symptoms more towards positive affect and life appreciation following MBSR (Hawtin and Sullivan 2011). Multiple studies have shown psychological distress improvements are mediated by daily home meditation practice time, a lack of which plausibly accounts at least in part for a lack of depression effect found in this meta‐analysis (Rosenzweig et al. 2010; Van Gordon et al. 2017) Given the GRADE very low certainty of effect finding versus active control, the true effect of MBSR on depression in FM is not clear.

Two of the included studies completed economic evaluations of MBSR. Pérez‐Aranda et al. (2019) completed cost effectiveness analyses illustrating improved quality adjusted life years and lower costs compared with the active control and treatment as usual group. Fjorback et al. (2013) also showed healthcare cost savings with reduced disability pension claims in the MBSR group, although the intervention group was five to six times more expensive than usual care in this study. The economic efficiency of MBSR in treating people with FM is consistent with research showing the cost effectiveness of MBSR in treating people with persistent lower back pain (Herman et al. 2017, 2020) and women with breast cancer (Lengacher et al. 2015).

This review has a number of strengths. The fixed effect model used for the meta‐analysis was appropriate given the homogeneity of the population being studied and specificity of the MBSR intervention (Deeks et al. 2023). Four of the 11 included studies had not previously been analysed in existing systematic reviews and meta analyses. Of these four, three have low or unclear risk of bias in all sections of the risk of bias tool, suggesting quality in addition to quantity data additions to the pooled outcome estimates. Despite this, all the pooled effect estimates were still based on relatively few trials, introducing serious imprecision in all but one effect estimate. The limited trials for each estimate also mean it is harder to interpret the reasons for statistical heterogeneity and near impossible to identify where publication bias may be impacting results (van Aert et al. 2019). Given the majority of trials included reported multiple outcomes of interest however, this somewhat negates the likelihood of publication bias, as trials confirming the null hypothesis in one outcome may still report a significant result in a separate outcome.

One notable limitation of the review is due to the prespecified review classification of long term follow, individual studies reported long term follow up time points ranging from 12 to 152 weeks after study commencement. The long term effect categorisation therefore spans an extensive amalgamation of time periods between 3 months to just under 3 years, making the analysis of overall effect longevity challenging.

For feasibility reasons, the review also only reviewed studies published in English, which may reduce the applicability of the findings to populations where English is not the primary language. Including relevant trials published in other languages in future reviews also has the potential to reduce effect estimate imprecision arising from analysing relatively few trials.

A further limitation is the vast majority of participants included in this meta‐analysis were female and most participants were also in their 40's and 50's. The results of this systematic review and meta‐analysis thus may not generalise to men or women of different age groups. The updated FM diagnostic criteria identify closer to 40% of people living with FM as men when biased recruitment is avoided (Wolfe et al. 2018) hence in the future trials should endeavour to recruit more men. The paucity of evidence on the effect of MBSR on males is particularly problematic given males with FM tend to endure pain for longer before seeking treatment (Conversano et al. 2021) and a greater proportion of men with FM live with depression (Henao‐Pérez et al. 2022).

Future research would benefit from investigating pain catastrophising alongside pain severity and exploring the interaction between the two, especially given the ease of administering a numerical pain rating scale outcome, as this would inform whether a subgroup of FM patients with particularly high pain catastrophising may benefit from MBSR due to a reduction in pain catastrophising and consequent drop in pain severity as shown in other FM research (Paschali et al. 2021; Angst et al. 2022). Concurrent investigation of neurobiological mechanisms using imaging or biomarkers could provide further clarity in future research, in addition to recording MBSR participant home practice time. Further high quality RCTs with gold standard control groups would provide valuable data for reducing imprecision and improving outcome effect estimates for all of the outcomes investigated in this meta‐analysis.

The findings from this meta‐analysis do not support the use of MBSR in FM for alleviating pain intensity, updating the findings from the last review exploring this question. Nonetheless, the findings do support the utilisation of MBSR as a cost effective, non pharmacological intervention for improving quality of life and pain catastrophising in FM, allowing some people with FM related pain to live with less suffering.

## Author Contributions

All authors contributed to the study conception and design. E.W. extracted data from the trials. K.H. and E.W. evaluated the inclusion and exclusion criteria and assessed the methodological quality of the trials. All authors contributed to drafting and revising the manuscript and approved this version to be published.

## Funding

E.W. received funding granted by City, University of London and Queen Mary's University of London in the form of a health advances in underrepresented populations (HARP) pre‐doctoral fellowship funded by Barts Charity. Funders had no direct role in the review.

## Conflicts of Interest

The authors declare no conflicts of interest.

## Data Availability

The data extracted from the included studies, the data used for analysis and the analytic code are available publicly at OSF here https://osf.io/tj5hx/files/osfstorage.
